# Registered nurses’ and older people’s experiences of participation in nutritional care in nursing homes: a descriptive qualitative study

**DOI:** 10.1186/s12912-018-0289-8

**Published:** 2018-05-10

**Authors:** Katarina Sjögren Forss, Jane Nilsson, Gunilla Borglin

**Affiliations:** 1 Department of Care Science, Faculty of Health and Society, Malmö University, SE-205 06 Malmö, Sweden; 2 Malmö Town, Borough Administration West, SE-214 66 Malmö, Sweden

**Keywords:** Care, Content analysis, Interviews, Malnutrition, Nursing interventions, Older people, Patient involvement, Registered nurse

## Abstract

**Background:**

The evaluation and treatment of older people’s nutritional care is generally viewed as a low priority by nurses. However, given that eating and drinking are fundamental human activities, the support and enhancement of an optimal nutritional status should be regarded as a vital part of nursing. Registered nurses must therefore be viewed as having an important role in assessing and evaluating the nutritional needs of older people as well as the ability to intervene in cases of malnutrition. This study aimed to illuminate the experience of participating in nutritional care from the perspectives of older people and registered nurses. A further aim is to illuminate the latter’s experience of nutritional care per se.

**Methods:**

A qualitative, descriptive design was adopted. Data were collected through semi-structured interviews (*n* = 12) with eight registered nurses and four older persons (mean age 85.7 years) in a city in the southern part of Sweden. The subsequent analysis was conducted by content analysis.

**Result:**

The analysis reflected three themes: ‘participation in nutritional care equals information’, ‘nutritional care out of remit and competence’ and ‘nutritional care more than just choosing a flavour’. They were interpreted to illuminate the experience of participation in nutritional care from the perspective of older people and RNs, and the latter’s experience of nutritional care in particular per se.

**Conclusions:**

Our findings indicate that a paternalistic attitude in care as well as asymmetry in the nurse-patient relationship are still common characteristics of modern clinical nursing practice for older people. Considering that participation should be central to nursing care, and despite the RN’s awareness of the importance of involving the older persons in their nutritional care this was not reflected in reality. Strategies to involve older persons in their nutritional care in a nursing home context need to take into account that for this population participation might not always be experienced as an important part of nursing care.

## Background

Registered Nurses (henceforth abbreviated ‘RN’) must be seen as an important player in assessing, evaluating and intervening in cases of malnutrition among older people. However, research shows that malnutrition in older people often goes unrecognised by RNs regardless of care context, for example, in nursing homes and/or hospitals [1, 2]. Although the prevalence of malnutrition is known to range from 15 to 40% in nursing homes [3–6], in terms of evaluation and treatment [7, 8], nutritional care in general is not a highly prioritised issue. This is noteworthy, as food and drink are essential human needs hence, a vital part of the fundamentals of care [9] and consequently a mandatory competency practice skill within the remit of the RNs role in care. Thus, the support and promotion of the optimal nutritional status of older people should be a prioritised area in nursing. A plausible explanation may be that RN’s responsibility within nutritional nursing care is unclear [10, 11]. Another explanation may be that RNs need further in-depth knowledge about nutritional nursing care issues [1], as this has been shown to increase their awareness of nutritional nursing care [12], and the nutritional status of older people living in nursing homes [13, 14].

Maintaining an optimal nutritional status is important for the wellbeing of older people and for promoting independent living [15]. However, despite this knowledge, malnutrition is a common and serious problem that contributes significantly to morbidity and mortality in this population group [16]. Malnutrition is associated with a decline in, for example, overall functional status as well as impaired muscle function and reduced cognitive function; in addition, malnutrition causes decreased bone mass, immune dysfunction, and anaemia [17]. The cause of malnutrition is often multifactorial and includes medical, physiological, psychological and social as well as environmental factors [16, 18]. Whereas the ageing process can often explain physiological and psychological factors, environmental factors in terms of meal ambiance can be easily modulated. It appears that environmental stimuli are not changed during the ageing process; therefore, environmental factors should always be considered as part of a nutritional nursing plan for older people living in nursing homes [19]. Given that the population of older people is increasing worldwide, it is crucial that healthcare professionals and RNs in particular actively work to prevent malnutrition and identify ways to meet the diet and nutrition needs of this vulnerable population.

It is reasonable to suggest that if RNs highlight and promote the importance of older people’s participation (‘participation’ and ‘involvement’ will be used synonymously in this paper) in their own nutritional nursing care, then the risk of malnutrition may decrease. Internationally and nationally there have been an obvious shift from the earlier hierarchical systems at the hospitals [20, 21] towards patients rights, which also encompass nursing. One plausible explanation for this change of focus in care are likely to be the strong democratic movements during the twenty-first century [22]. Resulting in that patients now, and regardless of context, are viewed as active participants in their care and healthcare decisions [23]. This shift is not attributed to changes in professional values alone. Rather, the move towards patient empowerment and person-centredness, is according to Christensen and Hewitt-Taylor [20] most likely the result of “….changes in the dominant views of society and lack of confidence in healthcare professionals, not simply because the healthcare professionals have adapted their thinking to be more respectful of patients’ rights ([20] p. 696).” In Sweden the patient’s right to be informed and to be made part of their own care is nowadays regulated by the Patient Act [24] and the Health and Medical Services Act [25]. Older people’s participation in all parts of their health care should therefore be considered as essential for healthcare professionals, particularly as a means to understand preferences and the optimisation of care [26]. Although previous research, conducted with a quantitative design, has shown that the involvement of older people has a positive effect on health outcomes, for example, in terms of health status, raising the energy intake [27] and satisfaction with care [28], older people are less often involved in their care than younger people [29, 30]. This despite studies that show older people want to be involved in their own care [31]. ‘Participation’ in care can mean to facilitate and encourage older people to share the responsibility of their own health and to support them in the decision-making process regarding their treatment and care [32] The involvement of older people in their own care is central for promoting person-centred nursing models, where RNs could play an important role in establishing this *modus operandi* in the care of older people.

Alharabi and colleagues [33] suggest that the lack of both understanding and confidence among healthcare professionals about involving older people in their nutritional care needs to be eliminated. The level of acceptance healthcare professionals has in regard to older people’s participation in care, has been shown to be influenced by the professionals’ need to maintain control and lack of time as well as the type of illnesses [34]. Further, ways to involve older people who reside in nursing homes in the food and meal activities appear to be limited [35, 36]. This may well be a factor that influences nutritional nursing care negatively in this population group. The majority of identified published nursing research seem to focus on the risk of malnutrition and its consequences either in the acute care setting or in the community alone, rather than older people’s experiences of participation in their nutritional care at nursing homes. Thus, in-depth knowledge about RNs, and older people’s experience of participation in nutritional care and the RN’s experiences of this type of care in a nursing home context is therefore still sparse. To the best of our knowledge, very few studies exist exploring this phenomenon in a nursing home context while including both older people and RNs. Therefore, this study aimed to illuminate the experience of participation in nutritional care from the perspective of older people residing at nursing homes and RNs. A further aim was also to illuminate the latter’s experience of nutritional care per se.

## Methods

A qualitative, descriptive design [37] was used to understand the experiences of nurses and older people who participate in nursing care when assessed as ‘at risk for malnutrition’ or as ‘malnourished’ from the informant’s point of view [38]. Data were collected through semi-structured interviews [37], and the subsequent analysis was inspired by Burnard’s [39, 40] description of content analysis.

### Study setting

This study took place in a city in the southern part of Sweden during spring 2016. In Sweden, the care of older people is primarily a public responsibility, and the provision of care and service for older people is mainly financed through taxes. The county councils are the regional providers of healthcare, but the municipalities are responsible for the care of older people living in their own homes or in nursing homes. The care is guided by RNs; however, the main providers of care and service in the municipalities are staff nurses and healthcare assistants. For the past 16 years in Sweden, the only way into the nursing profession has been through a degree programme, and since 2007, the only route has been via a bachelor’s degree in Nursing Science, which involves 3 years of study at university. The university education aims to equip the Swedish nursing students with the knowledge, competencies and skills needed to take the lead of care. Particularly the competencies needed to assess, diagnose, intervene and evaluate, in relation to the same essential human needs that was highlighted in care already by Florence Nightingale in the nineteenth century. Eating and drinking i.e. nutrition is hence part of the fundamentals of care [9], and a required practice competency expectation for nursing care also in Sweden.

Nursing homes are homelike residential care facilities for older people with mainly one-bed rooms that provide around-the-clock care. Residents at nursing homes are quite frail, and eight out of ten are aged 80 years or older. In the nursing homes, staff nurses and healthcare assisstants are on duty around the clock to provide regular care as well as palliative care. A RN (or, at times, two RNs depending on the size of the nursing home) is on call and accessible during officetime between Monday to Friday. Most residents have a primary care physician who is employed by the county council and who works at the local healthcare centre usually located near the nursing home where the resident lives.

### Sample and recruitment

In this study, 12 informants agreed to participate: The strategic sample [30] consisted of four older persons (65+), of which three were female and one was male. Our sample also consisted of eight RNs working in six different special accommodations. Of the RNs, two were male and six were female.

The inclusion criteria for the older persons were that they should be age 65 or older, cognitively intact, and able to read, speak and understand Swedish. They must also reside in one of the municipality’s nursing homes and have been assessed as ‘at risk for malnutrition’ or as ‘malnourished’ in accordance with the Mini Nutritional Assessment tool [41], which means they will have received a nursing diagnosis and intervention. Inclusion criteria for the RNs were that they should hold a permanent position at the nursing home, work at least 75% and be in charge of a ward not designated for older people suffering from cognitive decline.

#### Recruitment process

In the first step of the recruitment process, a sample of the city’s boroughs was made and nine out of 10 boroughs were contacted. For obvious ethical reasons, the borough in which the second author (JN) was working where not included in our study. After having received permission from the relevant branch heads to contact the municipality services, the coordinator of care was contacted. The latter contacted the patients’ responsible RNs in nine of the municipality’s nursing homes via email to inform them about the study. They acted as recruiters alone, and were asked to forward an invitation about participation in the study to older people and RNs meeting the inclusion criteria. In addition, the email contained an information letter about the study for the RNs. Six of the nine nursing homes that were contacted agreed to take part in the study. One reason given by the nursing homes for not wishing to participate was a lack of time.

The RN in charge of each nursing home facilitated the recruitment of the older persons. In total, seven older persons were assessed as meeting the study’s inclusion criteria and thus eligible for the study. Of these, four older persons with a mean age of 85.7 years (age range 74-90 years), residing in four different nursing homes, agreed to participate in the study (Table 1). Of the remaining three, one further individual initially agreed, but then withdrew at a later stage. Another became acutely ill, and the third withdrew due to feeling uninformed about malnutrition being the focus of the study. The interviewer provided written and verbal information about the study both when arranging the time for the interviews and before each interview began. All older persons gave written and verbal consent to their participation in the study.

**Table 1:** Characteristics – older persons

Code	Gender	Years in special accommodation	Body Mass Index [BMI]	Mini Nutritional Assessment [MNA scores]
A	Male	3	21.5	7 [malnourished]
B	Female	1	20.9	10 [risk of malnutrition]
C	Female	1	18.8	9 [risk of malnutrition]
D	Female	2	18	7 [malnourished]

The interviewer approached 14 eligible RNs, of which eight RNs with a mean age of 44.1 years (age range 28-67 years) accepted to participate (Table 2). Reasons given for not wanting to participate were, once again, a lack of time. During a face-to-face meeting, they were once again provided with verbal and written information, and a time and place was arranged for the interview. All informants in this study (*n* = 12) were informed about confidentiality and their right to withdraw at any point without needing to give any explanation for doing so.

**Table 2:** Characteristics – Registered nurses

Code	Gender	Work experience (years)	Educational level
E	Male	11	BSc Nursing
F	Female	3	BSc Nursing
G	Female	2	BSc Nursing
H	Female	4	BSc Nursing
I	Female	12	Diploma
J	Male	2	Diploma
K	Female	2	BSc Nursing
L	Female	43	Diploma

### Semi-structured interviews

**Fig. 1:**
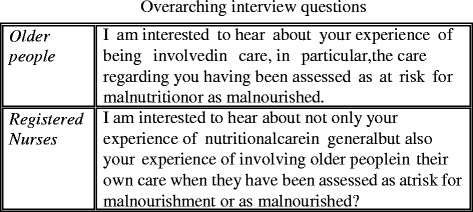
Overarching interview questions

The semi-structured interview method was used to collect the data [30], and they were conducted by the second author (JN) during April 2016. The interviews began with one overarching question (Fig. 1), which became more specific as the interviews proceeded. The overarching interview question was tested, by the second author (JN), on two RNs and one older person ahead of the interviews to assure its understandability and its relevance for the study aim. This test did not lead to any changes. Data from this test were not included in our analysis. Whenever clarification was needed during the interviews, general probing was used (ibid). The interviews with the older people were conducted in their home and at a time and place of their choice, while the interviews with the RNs took place during working hours in a separate room at the informant’s workplace – a place familiar to the informants and at a time that would maximise participation. The interviews lasted approximately 30–40 min and were tape recorded and transcribed before the analysis begun.

### Content analysis

The transcribed texts were analysed by a method influenced by content analysis, as outlined by Burnard [39, 40], and focused on both the manifest and latent levels, as outlined by Graneheim and Lundman [42] The process involved four steps. In the first step, the transcribed texts were read to gain an overall understanding and parts of the texts that were found to respond to the aim of the study were highlighted. The highlighted parts were in the second step condensed without losing the central meaning. In the third step, codes were created and in the fourth and final step, the codes were once again read, compared and contrasted with the text to ensure trustworthiness [39, 40]. In the final step, sub-themes involving several similar codes were also created and interpreted to represent three predominant themes relating to older people’s experience of being involved in their nutritional care as well as RN’s experience of older person participation and their experience of nutritional care per se. The second author (JN) was the main lead in the above described process of analysis. Additionally, the research team independently read and analysed the text and met regularly (approximately 1 h once every second week for about 10 weeks) to discuss and reach a consensus in all the different steps of the analysis. To further ensure the trustworthiness of the analysis, quotes from the informants are reported in the results.

## Results

Two of the themes, ‘participation in nutritional care equals information’ and ‘nutritional care out of remit and competence’ were interpreted to solely illuminate the RNs experience of older people’s participation in nutritional care and their experience of nutritional care per se. Whilst the third and last theme, ‘nutritional care – more than just choosing a flavour’ were interpreted to mirror older peoples experience of participation in their nutritional care.

### Participation in nutritional care equals information?

The theme, ‘participation in nutritional care equals information’, reflected how the RNs actually experienced older people’s participation in nutritional care to be equal with supplying them with information. The theme also reflected an awareness among the RNs about that the low level of patient participation in nutritional care. Especially when the older persons were as at risk of malnourishment or already diagnosed by the RNs as malnourished needed to be developed further as a part of their nutritional care. Thus, the main nursing strategy used to involve the older persons in their nutritional care was to offer some brief information to them. However, the RNs recognised that the information they offered to the older persons could be of better quality. This was experienced by the RNs as especially true for not only the information concerning the implications of being at risk of malnutrition or diagnosed as malnourished, actions to prevent it, and actions to remedy it, but also the information about the anticipated development, goals and nursing interventions targeting nutrition. An RN expressed this sentiment in her interview:

If we would have been better in our information, and included… yeah, particularly the different options. If we really had taken the time to go through it and to check, as there actually are many interventions…. trying to find the cause and then treat the actual reason. Yeah, I do not think we do it to the extent we should. [RN/H]

On the other hand, whether one should involve older people in their nutritional care or not stood out as an antipode. Particularly as the RN’s experiences also reflected that involving the older persons in his/her nutritional care was not at the top of the RN’s agenda. It also appeared unimportant to the RNs to inform the older persons about the outcomes of assessments performed, the actions to possibly take, and/or any interventions targeting their nutritional issue. The theme appeared more to mirror nursing directives and strict orders to remedy nutritional problems than strategies to support participation in care as a tool to reach common possible solutions in regards to the older persons nutritional care. The directives and orders were interpreted to be purely based on the RN’s experiences of what they thought was best for the older persons.

No, I do not do it [involve the older person] … no. But if I see that they are losing weight, then I visit them and tell them that I will order nutritional supplement drinks and that they should eat more. [RN/J]

Thus, this theme also reflected that, at times, the goal to involve older persons in their nutritional care departed from the common professional perspective of “I know what is best for you”:

It becomes a bit of an “I know best” attitude in this. That you might feel [like], ‘But I actually have this education and I do know this stuff, so therefore I choose this’. [RN/D]

Yeah. Thus, I think that one only thinks about it as any other treatment – that we think we know best about what works for them, and then we try that. [RN/K]

Involving older persons in their nutritional could be experiences as a challenge for some RNs. One particular challenge expressed as a barrier for participation in nutritional care became discernible when the older persons no longer experienced that their life was meaningful.

We try to involve them, but when they are at the point where they refuse [to engage] completely … they see no future and nothing, and they have lost all interest – then it is difficult. It is really very difficult to motivate [them and get them to understand] why food [eating] would make them feel fitter. [RN/L]

Experiencing a constant lack of time was also a barrier and meant that sometimes other types of nursing care was prioritised as more important than nutritional care. This, along with an underestimation of the older persons’ ability and willingness, were cited as reasons by the RNs to not involve older people in his/her nutritional care. One RN expresses this in plain terms:

Ugh, this is maybe an issue that is not a top priority, so one, yeah … yeah, one simply doesn’t have the time because we have other things to do here which one thinks is more important, but this is equally important. [RN/I]

Another experience by the RNs mirrored that the older persons did not always want to be involved in their nutritional care. Instead, the RNs experienced that they kicked the ball right back to the RNs corner. One RN expressed this as:

It depends on how interested the patient is. Some patients don’t want to know anything. If you start to talk about certain stuff, the reply is, “Yeah, yeah. You are handling it so well”. [RN/G]

The theme also indicated that both how well-informed the RN is about the older person’s general health and the RN’s perception of how independent and capable the older person was, were factors in the RN’s decision to involve the older person in their nutritional care or not.

I am not sure if they are well-informed enough to participate, and that is of course on us to give them that information and maybe offer some more suggestions for action than to arrive in their room and say, “Now we are doing like this”. I am bad at that [i.e. do the latter a lot]. [RN/H]

‘Participation in nutritional care equals information’ also illuminated certain insights concerning having missed out on an opportunity to engage and involve older persons in their own nutritional care. This was particularly mentioned as a lack of engagement in how the older persons experienced the mealtime ambience and the food served. One RN brought this up:

I think that this is something we are not so very good at, I think. I think that we do not really consider that, actually. They [the older people] are all sitting there in the dining room. It is, yes … No, this is something one can work on much more, actually. [RN/E]

Furthermore, the theme reflected that involving older people in their nutritional care actually was viewed by some RNs as a natural and integral part of professional nursing – particularly if the RNs perceived that the older person’s nutritional status were a vital factor for the older person’s general condition of health, as exemplified in the following quotation:

I usually inform that one has no energy, one cannot move around as much as one wishes. That the risk of attracting infections is bigger, and that one’s pain threshold is lower if one is malnourished. On the whole, one can withstand much if one is normally nourished, so to speak. [RN/F]

The theme additionally echoed a wish to allocate more time for troubleshooting and for understanding the underlying cause of the older people’s individual nutrition problems. The RNs felt that being able to depart from the root of the cause would facilitate older people’s participation in their nutritional care. This could be expressed as:

To inform them that there is a problem, what the alternatives could be to remedy it, and most importantly, why it is so important to not be malnourished – [to tell them] all the risks of being malnourished. [RN/K]

### Nutritional care out of remit and competence?

In the theme, ‘nutritional care out of remit and competence’, RNs experience of nutritional care per se was reflected as contradictory in many ways. How each individual RN experienced nutritional care per se was interpreted as not only having an impact on their performance of the nutritional nursing care on offer. Their experiences also seemed to resolve to what degree the RNs engaged in involving the older person in her/his nutritional care. The texts mirrored some RNs awareness of the importance to work proactively with nutrition in general and other RNs obliviousness, where nutritional care was not given much thought or refection. Those RNs reflecting a greater awareness of, and proactive engagement in nutritional care also tended to indicate knowledge about the relationship between nutritional status, older people’s health and their function expressed as:

One [the older person] has no strength if one doesn’t have enough food. One risks infections and sickness, and one has no resistance. Therefore, I put a strong emphasis on nourishment among old people. It should be frequent, small, calorie-dense portions and [served at] regular [times]: Three main meals and three in-between meals. One meal needs to be just before bedtime to avoid a long period of starvation at night, which will happen if one doesn’t eat just before bedtime. [RN/F]

In contrast, the oblivious approach to nutritional care was reflected upon by this RN:

One tries to ask if they have any particular likings in regards to food. I do not ask them much more than that. I actually do not think that much about that part – nutrition – at all. You see… there is… nature has to have its cause. In general, one eats… you know, one has less appetite for food and drink when getting older, and yes, that is how I view it in the bigger picture. [RN/I]

The theme, ‘nutritional care out of remit and competence’, also reflected experiences of insecurity and certain challenges, especially when it concerned what nursing actions and/or interventions to take when the older person was at risk for malnutrition. Some self-critical voices were apparent:

It’s most likely ignorance amongst the professionals, that is, by the nurse, um, yes. [RN/I]

Experiencing nutritional care as challenging could additionally mean that nutritional supplement drinks were the informants’ first and main choice of nursing intervention. It was also here where more self-critical voices were illuminated, and one informant expressed that the task of giving nutritional supplement drinks meant a job done and ticked off the list:

When we have given complete nutritional supplement drinks, we believe we have done it all. One can tick it off and record it … then one has done something. Is that not strange? [RN/K]

Another response which reflected this was made by another RN:

My initial thought is always complete nutritional supplement drinks … We met with the dietician yesterday and talked about this, and then he said that these [nutritional supplement drinks] are actually the last way out/option. One should focus more on adjusting the meal environment or use simpler methods to find out what is causing the person in question to not eat. [RN/H]

Despite interpreting the above statement to mean that teaming up with other healthcare professionals could result in different perspectives on nutritional care, the text reflected a rather cool and distanced relationship to for example the dieticians:

At times, I contact them [the dieticians], yes. …Yes, it can happen when there are problems with choking. It can happen when we do not [succeed with them] gaining weight despite us trying everything. …They might view it in a different way. … You get clever tips, but it is absolutely not always – it is [rather] seldom. [RN/E]

Another distanced relationship in nutritional care was also experienced in regard to the General Practitioner (GP). The informants’ overall experience was that not many “medical” actions could be done by the GP when an older person stops eating and drinking. Such issues had to be dealt with by the RNs, as this problem was situated within the domain of nursing. Here, another contradiction was reflected, as the texts mirrored both insecurity and challenges as well as experiences such as the RNs being much better equipped to handle nutrition involving older people and possessing more insight into how to deal with it despite being self-critical about the main nursing intervention i.e. nutritional supplement drinks.

I don’t deal with the GP as much. If I would have some bigger issues with a patient, [then] I would contact the dietician, but not the GP. I don’t really think they are much into this issue. [RN/F]

The theme ‘nutritional care out of remit and competence’ also reflected that the mealtime environment, mealtime ambiance and choice of what food served was not within the informant’s remit in nutritional care. The main control and responsibility for these factors had instead been handed down to the healthcare assistants, and this handing down of actual responsibility was expressed by two of the informants:

I think it is mainly [that] one has put it on the healthcare assistants [because] they are there all the time. It is them handling it, so it has been put down to them. [RN/J]

I am trying to engage, but it is mainly the enrolled nurse. It is their environment in the kitchen and so [on]. But if I think something is wrong, I do try to point it out – that they need to think about this or maybe that. [RN/G].

### Nutritional care more than just choosing a flavour?

In the final theme, ‘nutritional care more than just choosing a flavour’, the older persons’ experiences of participation in their nutritional care reflected that no easy standard approach exists to achieve a satisfactory level of involvement in their care. Some of the experiences reflected by the RNs in the theme ‘participation in nutritional care equals information’ also mirrored the older persons’ experiences. This was especially reflected amongst those older people who experienced being uninformed about what could be done or which care could remedy their nutritional status. One older person states this plainly:

No one tells you anything here. They do what they want … that is how it is. [OP/D]

Additionally, the older persons’ experience of not being involved or having the possibility to discuss things with the RNs about how the nutritional care was to be planned indicated frustration, and this came through in one plain-speaking older person’s interview could be expressed as:

One is not allowed to decide anything. [OP/C]

Other experiences were also recounted, as some of the older persons perceived that they had been informed about what malnutrition could mean when being older. They could describe how being malnourished affects their energy levels and is detrimental for their health. One informant expressed it in the following admission:

It was okay. Yeah, one could say it was good [the information about being malnourished]. … I needed to eat more. [OP/A]

In these cases, despite having been informed by the RN, the older persons perceived that they not had been involved in planning the treatment meant to target their malnourishment or risk of malnutrition. These older people experienced that they were passive receivers of the nutritional care and the information given.

When malnourished, no, we did not [participate]. I got nutritional supplement drinks from the nurse. I got Actimel. [OP/A]

The theme ‘nutritional care more than just choosing a flavour’ also suggested that when having been assessed as at risk of or as malnourished the only nursing action for this was to instruct the older persons to drink supplemental nutrition drinks, and the older person’s only involvement was to be allowed to “pick the flavour you like”. The nutritional supplement drinks were given until the individual’s weight was back up to a healthy level.

For a while, I got these … bottles. I got one three times a day, such bottle [nutritional supplement drinks]. But now, I am told by them that I don’t need them anymore. [OP/B]

Having been slim and slender throughout life and consequently having a low BMI since youth meant that some older people experienced nutritional care and weight gain as an upsetting ordeal. Emotions such as nervousness and anxiety about weight gain and a changed body image were expressed. This experience was corroborated by the RNs, who perceived that some older persons took on a highly passive role in regard to involvement and were not particularly adherent to the action plan purely based on anxiety about gaining too much weight.

I am eating and gaining weight. Now I am scared to gain far too much weight. [OP/B]

In stark contrast to the one nursing intervention on offer (i.e. nutritional supplement drinks) was the older persons’ views reflecting the possibility to decide his/her meals. On one hand, the older person would experience a boost in energy by the drinks, but on the other hand, they found them difficult, as they quickly felt full with them. If they were given a choice, the older people preferred normal food over drinking more than one nutritional supplement drink a day.

They tell you that you can drink more in one day, but one can’t. It is that much … one cannot drink four or as much as possible. So, so that is how it is … for me, there is a limit of one bottle [nutritional supplement drink] a day. And that is not enough to go up…. [in weight]. [OP/C]

The theme ‘nutritional care more than just choosing a flavour’ also entailed the older person’s experiences of involvement in the mealtime environment and the setting of the daily food menu. They expressed a wish to be involved in both the shaping of the environment and the menu. One older person expressed her frustration:

And I thought, ‘It is only this time [that] it is a catastrophe. It is only this time … and not tomorrow’. Oh, yes, I am put at the same place by the table every time [next to a resident she does not want to be near]. [They think,] ‘She is so kind, and she is so tiny, so it doesn’t matter, [so] we place her here’. [OP/C]

The food on offer at the nursing home was not experienced as highly rated, and when asked the direct question of whether the older persons experienced any possibility to become involved in the food that was served, two of the informants gave a reply indicating that the menu was not able to be changed:

I get the menu, and then [I] have to eat [what’s on it]. [OP/A]

Nothing [involving participation with food]. They only serve it. On the table in front of me. [OP/D]

The theme reflected that breakfast was the only meal where the older persons experienced that they could be involved. Breakfast food was presented on the table, and one could choose whatever they liked. The older persons perceived that they could ask for something else (i.e. extra outside ordinary mealtimes and the set menu), but otherwise their involvement was also limited here. The theme reflects the wish to be involved in the menu as well as the insight that if the food tasted a bit better, then they would automatically eat more. However, despite not experiencing any meaningful involvement in their nutrition, the food served or mealtime ambience, the older persons still felt that the staff tried to accommodate as many of their wishes as possible.

I would like to decide my food and [choose] food that is good. Good gravy. That the food is … when you get pork shops or pork shoulder. Then you get no gravy. They remove the best part. I would like to have my mum’s great steaks [giggles]. I know I cannot get it. [OP/C]

Coffee and tea – one can have at any time you go down and say, “Now I fancy a cup of coffee” [then they say,] “I will arrange that for you”. They are good in that sense: “Have all of you got [coffee or tea]?” [OP/C]

All meals were served at set times, but they could be slightly moved for the unique individual. This was perceived as some degree of being involved as well as being in control in some way. One older person explains:

We have our set mealtimes here: Breakfast at 8 o’clock, dinner at 12 o’clock, coffee at 2 o’clock, and supper at 5 o’clock. This is how it is every day. But if you are doing something, then they save your food and reheat it for you when you arrive. [OP/B]

Lastly, the theme reflects that the older persons would experience the need to accept what was laid out on the table, as they perceived that it would not be right to complain about the food being served. One of the older people explains:

If I don’t want it, they can arrange something else. But I never do that. I am ashamed of doing that. They do all they can to prepare good food, and then how it comes out – that’s another matter – but one has to try to eat it. [OP/A]

## Discussion

The analysis indicate that RN’s experience of older people’s participation in nutritional care and their experiences of nutritional care per se could be understood in the light of two predominant themes: ‘participation in nutritional care equals information’ ‘nutritional care out of remit and competence’. While the older peoples experience of their participation in their nutritional care could be understood in the light of the theme, ‘nutritional care more than just choosing a flavour.’

The lack of engagement from the RNs regarding involving the older persons in their nutritional care stood out as noteworthy in the theme, ‘participation in nutritional care equals information’, particularly as the concept of *person-centred care* is cited as one of healthcare’s main priorities today, but the concept is also one of the six core competencies [43] RNs are suggested to base care on. Our findings put forward that the RNs saw participation as analogous to simply informing the older persons about their nutritional status. When taking the time to reflect, the RNs experienced that the information they gave could have been better; however, a lack of time was cited one barrier for giving information. Our findings also revealed that RNs seemed to prefer to give directives and strict orders rather than engage the older person in a discussion about what could be the best ways to improve their nutritional status. However, our findings may not be unexpected, given that Longtin and colleagues [34] found that the staff’s acceptance of patients’ participation in care is affected by their need to preserve control and by a lack of time. Factors entrenched in the context of care such as a task-oriented practice (e.g. giving directives) is also known to obstruct participation [44]. Others [35, 36] have shown that the participation of older persons, particularly those residing in nursing homes like those of this study, are rarely, if at all, involved in activities relating to nutrition or nutritional care.

To involve older people in their own care and inform them adequately requires effective communication. However, research into participation in clinical practice found that although RNs speak about the importance of communication, they were only observed to have contact with the persons they cared for when they had a task to complete [44, 45]. This knowledge has implications, particularly in the Swedish context of nursing homes, as the daily care is delivered by healthcare assistants and not by the RNs. The latter are mainly called upon in unexpected care events; thus, this does not leave many natural interaction points between the RNs and the older persons. It is clearly important to acknowledge that adequate information [46] is vital in supporting people to participate in decision-making about their own care, especially as information is central for the patient’s ability to make decisions about their care. In particular, when the information given is used as a tool to support the RN’s agenda, it becomes ostensible rather than a factor that leads to true participation. We recommend that participation in care has to be more than simply informing the older person about directives and already planned actions. Collaboration and the sharing of power between the RN and the older persons appear just as important as offering them relevant information. Thus, our findings imply, despite that patient participation in care is regulated by law in Sweden, that nutritional care for older people residing in nursing homes may yet have some way to go before participation becomes a natural part of nutritional care in this context. There are no easy solutions to remedy this; however, a person-centred care approach should be central in the nutritional care of older people.

Involving older persons in their care appears to occur when RNs possess knowledge and awareness about the components of nutritional care as reflected in the theme ‘nutritional care out of remit and competence’ This stood out in stark contrast to some RNs views which suggested nature needs to have its place in old age. It also contrasts with certain RNs feelings of insecurity and frustration about nutritional nursing care and strategies for involvement. Despite the latter and that support was available both in the form of the GP and dietician, nutritional supplement drinks were shown to be the nurses’ gold standard for those older people assessed at risk for malnutrition or as malnourished. It is clear that knowledge and competence support RNs in involving older persons in their care. However, the ease at which RNs confess to lack sufficient competence in nutritional care and how to involve the older persons in this care is unexpected, although others have also highlighted shortcomings in nutritional nursing care. For example, Alharabi and colleagues [33] suggest the need to remove the lack of understanding and confidence among healthcare professionals when it concerns how to involve older people in their nutritional care. Suominen and colleagues [1] further support this by putting forward that RNs need more in-depth knowledge about nutrition issues to raise their awareness about nutritional nursing care. This corroborates with our interpretation of the connection between RN’s knowledge and awareness of nutrition and the RN’s level of involving patients. Moreover, interprofessional collaboration in addition to the collaboration between RNs and older persons is required to establish appropriate and sustainable nutritional care.

Considering that one of the core competencies for RNs put forward by Cronenwett et al. [43] is that nursing care on offer should be evidence-based, it was rather unexpected that nutritional supplement drinks were the first line of nursing intervention in treating older people assessed at risk for malnutrition or as malnourished. Especially, considering the weak evidence about their actual effect on malnutrition i.e. effect in weight gain or improved function among older people [47, 48]. It seems reasonable to draw the conclusion that nutritional care in this context seemingly still not rests on an evidence-based care. Taking this into account, it becomes difficult to whitewash or give alternative interpretations to the kind of nutritional nursing care reflected by statements such as “a job done and ticked off”. Understanding and applying evidence-based nutritional knowledge are important ways to effectively assess dietary intake and provide appropriate guidance, counselling and treatment to older persons. This must be vital despite the fact that a recent systematic review [49] aiming to determine the effect of nursing interventions targeting fundamentals of care [9] such as nutritional care concluded that current evidence for nutritional nursing care interventions was sparse, of poor quality and unfit to provide evidence-based guidance to RNs in clinical practice. Consequently, educational efforts alone will not be enough to improve nutritional care as Richards et al. [49] highlights that nursing research additionally need to step up when it concerns effective nursing care interventions targeting fundamentals of care.

Although the RNs ultimately are responsible for care, thus expected to take on the role as the point-of-care leader, our findings indicated that some of the responsibilities for the older persons nutritional care depended upon the health care assistants. Leaving some of the RNs with the experience that some of the general nutritional care were out of their remit. On the other hand, RNs were quite determined that the GPs not were in a position to support nursing care in case of nutritional challenges i.e. older patients refusing to eat and drink as this belonged to the domain of nursing. It is not based on these findings reasonable to predict the reasons for these contradictory experiences. We know that RNs providing leadership at the point-of-care can have a positive impact on clinical practice but also introduce leadership behaviours of importance for all roles [50]. However, to be able to lead care demands competent and safe RNs and, our findings did at time, reflect experiences of both insecurity and lack of competency concerning nutritional care. Additionally, teamwork is known to improve patient planning, is clinically more efficient and supports a person-centred care [51]. Working in interprofessional teams in order to ensure continuity of care, patient safety and quality of care is also one of the six core competencies suggested for nurses to possess to meet health care standards [43] and would be a realistic approach to improve older peoples nutritional care in nursing homes.

Older persons’ participation in their own nutritional care appears to be an ambiguous and tortuous path. In the theme, ‘nutritional care more than just choosing a flavour’, it is clear that, although the older people wished to be involved in both what was on the menu and in shaping the environment, their involvement was restricted to choosing what they wanted for breakfast and what flavour they wanted if they required a nutritional supplement drink. No easy standard approach exists to achieve a satisfactory level of involvement in their own care. The older people stated that communication often failed, and they were not able to be involved or have the possibility to discuss things over with the RNs about how their nutritional care was planned. Our findings are corroborated by others. Say and colleagues [26], conclude in their literature review that it is important to remember that participation may not be acceptable or appropriate for everyone. In a qualitative study by Nyborg and colleagues [52] the older people experienced difficulties when participating (i.e. their involvement in decisions and in their own care). The reason given by the older persons in the study was their deteriorating capability to do so. Additionally, an older person’s desired level of participation in his/her care may be influenced by their generational values. This notion falls in line with Nyborg and colleagues [52], who state that today’s participation ideology is based on individualism, which they suggest is likely to conflict with the current older generation’s “commonly held values of solidarity and community” (p.1). Consequently, research [53] has shown that older people’s gratitude for the healthcare system and to healthcare staff at times overshadows not being informed or involved; that is, they reject their own needs and preferences. Another study by Penney and Wellard [54] found that participation in care was equated with being independent by the older persons. The later can be extra challenging for the RNs to handle when engaging in strategies promoting participation, as it is likely that older people residing in nursing homes already experience that their independence is restricted. Nursing that strives to involve older persons in their care and decisions about their care would therefore gain from departing from person-centred care models, particularly as is seems fair to assume that these kinds of nursing models automatically mean acceptance of the RNs to offer a care adapted to what degree the older person wishes to participate.

### Study limitations – Strength and weaknesses

Our study design allowed an emphasis to be placed on the statements and interpretations of those being studied. However, its relatively small sample (*n* = 12) has implications for the trustworthiness of the findings. Our sampling technique ensured the possibility of capturing different views and perceptions, and although the sampling was strategically conducted [30], it was relatively homogenous in aspects like gender when it concerned the RNs. This implies that the result can be specific for female RNs within this context and that homogeneity can affect the transferability of the result. However, their heterogeneous ages, levels of education and amount of time in their position, in addition to including two RNs who are male, may counterbalance this. It was a challenge to recruit older people, and the sample size ended up being restricted to four informants (three women and one man), as three informants chose to withdraw from the study prior to the interviews. Therefore, due to the small sample size and the homogeneity, generalisations must be made with caution. However, to the best of our knowledge, few studies regarding older people’s experiences of involvement in nutritional care have been conducted, and thus, this study contributes with new knowledge to this field.

The amalgam of realities presented here may be regarded as the views of the informants, and as such, may be transferable to similar settings. Finally, the risk of subjectivity in the data interpretation always exists, as there is always more than one possible way to interpret a text. Our method of analysis – content analysis [35] – allows the possibility to justify the texts by structuring and presenting them using categories and themes. However, the risk of subjectivity always remains, as data interpretation can be influenced by the interpreter’s life experience and ability [55]. To reduce this risk and to enhance the credibility of this study, the authors worked together throughout the phases of analysis to strengthen the interpretations, not by achieving consensus or arriving at identical formulations in interpretations but by supplementing and contesting each other’s readings. By describing the analytical procedure used and presenting quotations from the interview texts, we have hopefully enabled the reader to consider the interpretation valid [56] and trustworthy. A qualitative study such as this is limited in regard to its transferability and its relevance to other types of settings; consequently, this should be taken into account when evaluating our findings.

## Conclusion

Our findings are somewhat disheartening because today substantial existing knowledge confirms that it is important for healthcare professionals to strive towards true participation in care. However, we acknowledge that participation is a complex process which requires all factors to work together well before participation and decision-making become a natural part of essential care for older people. Although our findings suggest that RNs should be aware of the importance of involving older people in their care, they also indicate that, despite the knowledge that participation should be central to nursing, this has yet to become a reality despite its regulation by law. Therefore, educational strategies that support RNs in developing the competences needed to enable older people’s participation in nursing care should be a priority, even during the first years of nurses’ training. Furthermore, our findings indicate that a paternalistic attitude in care as well as asymmetry in the nurse-patient relationship are still common characteristics of modern clinical nursing practice in the care of older people. However, we envisage that this unsuccessful approach in care will be phased out when new cohorts of older people such as baby boomers become the new consumers of health and social care.

Nursing care striving to involving older people in their own care, and those making the decisions need to be aware that different strategies may be needed to make participation a reality. Although our findings may need to be interpreted with caution, they reflect that getting involved in care was not always as high on the older persons’ agenda as one might expect. Thus, developed strategies for participation need to take this into account. A person-centred approach to care could be one model of care to facilitate this. To be successful in this work, an interdisciplinary approach including both RNs, GPs and Health Care assistants is needed. To promote effective collaboration, it is important to assure that the roles of the team members is clear, but also that RNs are ready to take on the role as the point-of-care leader. Enhancement of collaboration by communicating roles and making work agreements should therefore be continuously on the agenda. It is also important find strategies to empower RNs to take the point-of-care leadership for the fundamentals of care and here especially for nutritional care. However, before doing this it seems that there is a need to raise competence and knowledge as our findings indicate both insecurity and lack of competence among RNs concerning nutritional care.

Finally, it appears important to conclude by suggesting that different strategies are needed for researchers to be able to explore more in-depth how participation can be achieved or experienced among older people, particularly those residing in nursing homes, as this is an extremely vulnerable group. For them to participate in and be able to decide about their care may be effective ways to support the older person’s feelings of independence and well-being.

It is important to get a deeper understanding of what matters to patients and to RNs who are responsible for deliver nutritional care in an often complex and challenging environment, and more research in the field is needed. Nutritional care must be based on evidence and future research also need to focus on the effectiveness of fundamentals of care to support nursing knowledge in the deliverance of an evidence-based care.

## Abbreviations

GP: General practitioner
RN: Registered nurses
